# Antioxidant Intervention against Male Infertility: Time to Design Novel Strategies

**DOI:** 10.3390/biomedicines10123058

**Published:** 2022-11-28

**Authors:** Cristóbal Ávila, José Ignacio Vinay, Marzia Arese, Luciano Saso, Ramón Rodrigo

**Affiliations:** 1 Molecular and Clinical Pharmacology Program, Institute of Biomedical Sciences, Faculty of Medicine, University of Chile, Santiago 8380000, Chile; 2 Urology Department, University of Chile Clinical Hospital, Santiago 8380000, Chile; 3 Andrology Unit, Shady Grove Fertility, Santiago 7650672, Chile; 4 Department of Biochemical Sciences “A. Rossi-Fanelli”, Sapienza University of Rome, 00185 Rome, Italy; 5 Department of Physiology and Pharmacology “Vittorio Erspamer”, Faculty of Pharmacy and Medicine, Sapienza University, 00185 Rome, Italy

**Keywords:** male infertility, oxidative stress, reactive oxygen species, spermatozoa, mitochondrial dysfunction, antioxidants

## Abstract

Infertility is a highly prevalent condition, affecting 9–20% of couples worldwide. Among the identifiable causes, the male factor stands out in about half of infertile couples, representing a growing problem. Accordingly, there has been a decline in both global fertility rates and sperm counts in recent years. Remarkably, nearly 80% of cases of male infertility (MI) have no clinically identifiable aetiology. Among the mechanisms likely plausible to account for idiopathic cases, oxidative stress (OS) has currently been increasingly recognized as a key factor in MI, through phenomena such as mitochondrial dysfunction, lipid peroxidation, DNA damage and fragmentation and finally, sperm apoptosis. In addition, elevated reactive oxygen species (ROS) levels in semen are associated with worse reproductive outcomes. However, despite an increasing understanding on the role of OS in the pathophysiology of MI, therapeutic interventions based on antioxidants have not yet provided a consistent benefit for MI, and there is currently no clear consensus on the optimal antioxidant constituents or regimen. Therefore, there is currently no applicable antioxidant treatment against this problem. This review presents an approach aimed at designing an antioxidant strategy based on the particular biological properties of sperm and their relationships with OS.

## 1. Introduction

Infertility is defined as the failure to achieve a spontaneous pregnancy despite 1 year of practicing regular sexual intercourse without a contraceptive [1]. It affects 9 to 20% of all couples [2] and a male factor is an identifiable cause in 50% of infertile couples [2,3]. Furthermore, current evidence suggests a decline in global fertility rates worldwide from 4.7 to 2.4 live births between 1950 and 2017 [4]. In addition, there has been a parallel decline in sperm counts (0.70 million/mL/year) observed between 1981 and 2013 [5].

Identifiable causes of male infertility comprise as few as 10% of the total cases, with around 5% being sperm transport disorders [6] and the other 5% being endocrine and systemic disorders with hypogonadotropic hypogonadism [6,7,8,9]. Most infertile men fall into two categories: primary testicular defects in spermatogenesis (65–80%, with the majority of these cases being idiopathic dysspermatogenesis, an isolated defect in spermatogenesis without an identifiable cause) and idiopathic or unexplained male infertility (15–30%) [10], in which an infertile man presents with normal seminal fluid analysis and no apparent cause for infertility [11].

The redox state of a biological system can be defined as the balance between its oxidative elements and the capacity of its antioxidant elements. The main oxidant elements are reactive oxygen species (ROS), a group of highly reactive oxidant agents, including radical anion superoxide, hydrogen peroxide, hydroxyl radical and singlet oxygen. At low concentrations, ROS are actually essential for normal cell homeostasis and are involved in various cell signalling pathways [12]. Conversely, when antioxidant systems of the cell are overwhelmed by ROS concentrations (a state known as oxidative stress [OS]), oxidation of biomolecules can occur. Consequently, resulting pathophysiological events can range from cell dysfunction to cell death [13]. Thus, ROS have been identified as a key factor in a variety of pathologies such as atherosclerosis [14], arterial hypertension [15], atrial fibrillation [16], heart failure [17], stroke [18], metabolic syndrome [19], NAFLD [20], Alzheimer’s disease [21] and other neurodegenerative diseases [22], among others.

Despite its epidemiological relevance and its widely accepted association to OS, no antioxidant strategy has shown consistent benefit on male infertility. This review focuses on the role of OS in idiopathic dysspermatogenesis and unexplained male infertility, and new approaches on the development of therapeutic strategies against male infertility.

## 2. Relationship between Male Infertility and Reactive Oxygen Species

In 1994, Aitken proposed the loss of sperm function subsequent to the induction of peroxidative damage by ROS originating from the spermatozoa as a theory for male infertility [23]. Since then, there has been a growing body of evidence pointing to OS as a key phenomenon in the pathophysiology of male infertility. A recent update on male infertility by the European Association of Urology discusses the increasing evidence suggesting that in the setting of unexplained infertility (men with normal sperm parameters but unable to conceive with a healthy female partner) can present abnormalities at the molecular level, such as increased sperm DNA fragmentation (SDF) [24,25,26] and elevated seminal reactive oxygen species (ROS) [26], which appear to result in worse reproductive outcomes in couples [24,26,27,28,29,30,31].

Before further exploring the pathophysiological mechanisms behind the relationship between OS and male infertility, it is imperative to properly characterise the oxidant and antioxidant elements playing in the particular setting of human spermatozoa. 

## 3. ROS and Sperm Physiology

The cellular oxidative homeostasis in spermatozoa relies on two groups of components that interact in maintaining this balance: ROS and antioxidants.

Among reactive oxygen species that standout within the biology of spermatozoa are (1) superoxide radical anion (O_2_^−^), primarily originating in mitochondria via the slippage of an electron from the electron transport chain (ETC) to molecular oxygen during oxidative phosphorylation, (2) hydrogen peroxide (H_2_O_2_) and the (3) hydroxyl radical (·OH) [32].

Main endogenous ROS sources include: (1) mitochondrial ETC and (2) membrane-associated reduced nicotinamide adenine dinucleotide phosphate (NADPH) oxidase. Of the aforementioned, the main endogenous source of ROS is the latter [33]; the electron flux through the ETC, located in the inner mitochondrial membrane, is instrumental to oxidative phosphorylation and ATP synthesis. Despite being a highly efficient process, 1–2% of oxygen is reduced to O_2_.^−^ by single electron transfer mediated by complexes I and III of the ETC [34]. Hydrogen peroxide levels are also a product of this phenomenon, given the high activity of superoxide dismutase 2 (SOD2) in mitochondria, which dismutates O_2_^−^ to H_2_O_2_ [35]. Meanwhile, hydroxyl radical is a product of the reaction of transition metals both with superoxide anion (Haber-Weiss reaction) [36] and hydrogen peroxide (Fenton reaction) [37].

Although NADPH oxidase is expressed in human spermatozoa in the form of NOX5 [38], the real impact of its activity both in physiological and pathophysiological settings is unknown [39]. Moreover, there is evidence showing that it lacks significant activity [40] and that the ROS producing activity of NOX5 is significantly lower than that of the NADPH-oxidase found in leukocytes [41]. Alternatively, it could be argued that NADPH oxidase found in leukocytes is a relevant source of ROS in sperm. Aitken & West conducted a study analysing the relationship between ROS production and leukocyte infiltration in 109 samples of human ejaculate from non-selected volunteers. Interestingly, 6 cases showed an association of elevated ROS production and leukocyte concentrations. However, in the majority of cases exhibiting high ROS production, leukocyte numbers were low or absent, suggesting that the relative contribution of leukocytes to ROS production in sperm is low [42]. Furthermore, the author propounds that, under in vivo conditions, the induction of OS on spermatozoa by infiltrating leukocytes is unlikely given the protective properties of seminal plasma [23].

Additionally, there are several environmental risk factors for male infertility whose deleterious effect over spermatozoa is attributable to their role as exogenous ROS sources, namely: obesity [43,44,45], smoking [46,47,48,49], alcohol use [50], ionising and non-ionising radiations [51], air pollution [52], among others.

As previously outlined, ROS are essential to proper cell functioning under physiological conditions, and male germ cells are not the exception. Low ROS levels are crucial for the acquisition of sperm functions during the final step of development of the mature spermatozoa. Each of the following processes take place as the spermatozoa transport through the female reproductive tract:

- Capacitation: Spermatozoa undergo molecular modifications such as basification of intracellular pH, activation of c-AMP dependent pathways, removal of cholesterol from sperm membrane, and phosphorylation of serine, threonine and tyrosine residues in proteins [33]. Capacitation has been shown to be suppressed when spermatozoa are incubated with catalase, which is consistent with the finding that low levels of H_2_O_2_ result in a higher rate of capacitated spermatozoa [53]. Low levels of ROS have been shown to positively modulate several pathways involved in the molecular modifications in the capacitation process [54].
- Hyperactivation: Hyperactivation is considered a subprocess of capacitation. When reaching the oocyte, spermatozoa exhibit a high amplitude, nonlinear flagellar movement which is instrumental to the impulse of spermatozoa through cumulus cells surrounding the oocyte. O_2_^−^ concentrations seem to trigger this phenomenon [55]. Higher degrees of SOD activity were shown to block hyperactivation induced by O_2_^−^ in semen samples [56].
- Acrosome reaction: Consists of exocytosis of the acrosomal matrix rich in digestive enzymes such as acrosin and hyaluronidase, essential for sperm penetration across the cumulus cells and zona pellucida. Capacitation is a necessary condition for this process to take place. Low concentrations of O_2_^−^ and H_2_O_2_ have been shown to possess a role in acrosome reaction [57,58].
- Sperm-oocyte fusion during fertilisation: Membrane fluidity of spermatozoa is crucial for sperm-oocyte fusion. In turn, membrane fluidity is influenced by polyunsaturated fatty acid (PUFA) content and phospholipase A2, whose activity is upregulated by O_2_^−^/H_2_O_2_-dependent kinase activation [59,60].

## 4. Antioxidants and Sperm Physiology

Conversely, antioxidants can be broadly categorised into two types: enzymatic and non-enzymatic. The three most relevant enzymes of the antioxidants system in semen are superoxide dismutase (SOD), catalase (CAT) and glutathione peroxidase (GPX) [47].

SOD activity is significant both in spermatozoa and seminal fluid, being considerably higher in the latter when compared to other extracellular fluids. Three isoforms of this enzyme have been identified in mammals: SOD1/CuZn-SOD (cytosolic), SOD2/Mn-SOD (mitochondrial) and SOD3/EC-SOD (extracellular) [61]. SOD dismutates O_2_^−^ into molecular oxygen and H_2_O_2_. Due to its location, SOD2 seems particularly critical in the context of human spermatozoa since increased SOD2 expression is associated with a reduction of mitochondrial O_2_^−^ levels, attenuated age-dependent increase of oxidative damage, and better preservation of mitochondrial function [62,63,64].

GPX comprises a group of enzymes in charge of catalysing the reduction of H_2_O_2_ to oxygen and water. The most relevant isoforms in spermatozoa seem to be GPX1, GPX4, and phospholipid hydroperoxide glutathione peroxidase (PHGPx, which uses selenium as cofactor), playing a role in the protection of structural integrity, motility and viability of sperm [65,66].

CAT catalyses the conversion of H_2_O_2_ to molecular oxygen and water. A constant, low level of activity of this enzyme has been described in the testicles of rats [67]. Furthermore, lower levels of seminal CAT have been found in asthenozoospermic men when compared with normozoospermic men, suggesting the relevance of this enzyme in male fertility [68].

Non-enzymatic antioxidants can also be endogenous or exogenous, being the most relevant in spermatozoa: glutathione, selenium, carotenoids such a lycopene, ascorbic acid and α-tocopherol, which exert their antioxidant effects through direct neutralisation, promoting expression of antioxidant enzymes, or acting as cofactors for antioxidant enzymes.

In a 2011 study by Moretti et al., the total antioxidant power of seminal plasma was estimated to be ten times higher than that of blood and comprising a combination of small-molecular-mass free radical scavengers, including vitamin C, uric acid, tyrosine, unspecified polyphenols, reduced glutathione, and hypotaurine. Additional small-molecular-mass scavengers, such as vitamin E, are also present in seminal plasma [69].

This schematic division of elements in the antioxidant system is for the sole purpose of simplifying their characterization: indeed, these elements are intertwined and work synergically.

It is interesting to note that, since low levels of ROS are necessary during processes such as capacitation and acrosome reaction, the overuse of antioxidants could be deleterious due to reduced ROS generation and intracellular concentration [70]. This excessive administration of antioxidants could shift the redox balance of the cell in favour of a predominantly reductive state, known as reductive stress (RS) [71], which could even result in impairment of spermiogram variables such as concentration, morphology and motility [70]. Moreover, vitamin C and vitamin E, both widely used in clinical trials for male infertility, have shown detrimental effects in the context of fertility [72], such as increased sperm decondensation in men treated with vitamins C and E, β-carotene, selenium and zinc [73]. Further research is needed in order to describe the optimal antioxidant doses, but adverse effects could be related to excessive dosing and lack of understanding of the synergistic effects of antioxidant compounds [74].

## 5. Role of ROS in the Pathophysiology of Male Infertility

In a 2021 review paper, Ritchie et al. described mitochondrial dysfunction, lipid peroxidation, DNA damage and finally, apoptosis as the main phenomena by which OS produces its deleterious effect over spermatozoa morphology and function [32]. 

**Figure 1:**
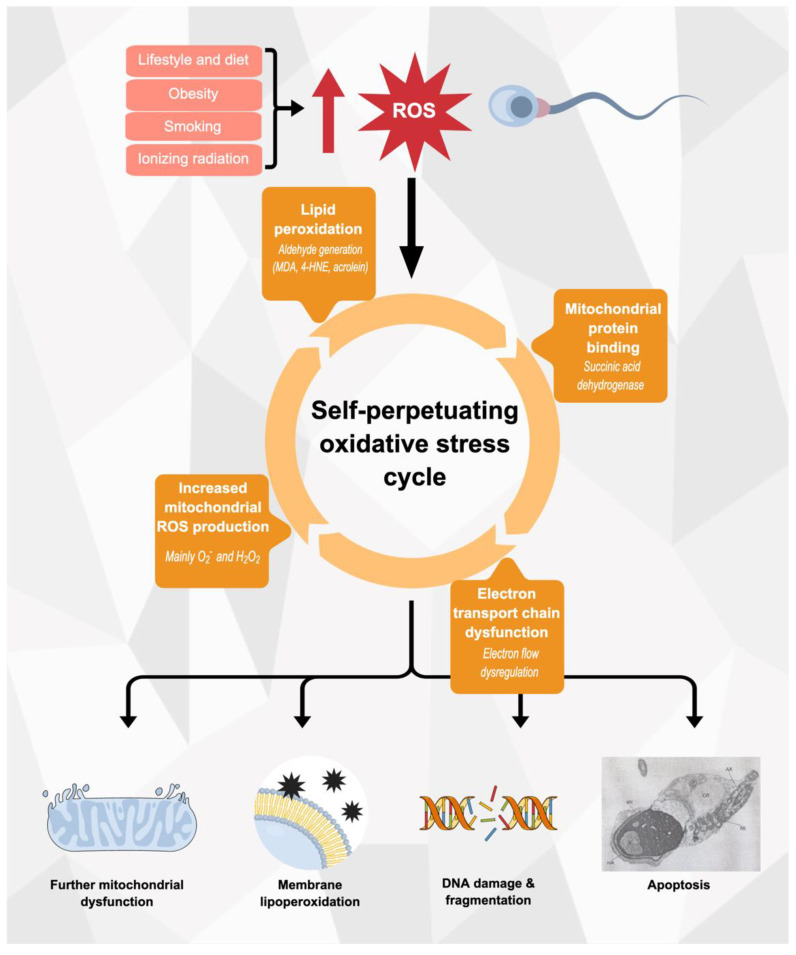
A number of environmental and lifestyle factors have been linked to increased levels of ROS, being the possible starting point of OS in spermatozoa. Regardless of their origin, an increased ROS concentration could be the driving factor for the establishment of mitochondrial ROS production, resulting in a self-perpetuating cycle that ultimately produces several cellular dysfunctions and constitutes the basis of the pathophysiology that underlies the association between ROS levels and male infertility. MDA: malondialdehyde; 4-HNE: 4-hydroxynonenal. This figure is the author’s original work using Mind the Graph Scientific Illustrator.

Alternatively, Aitken and colleagues assign a key role to mitochondrial dysfunction as the initiating event, from which the rest follow: cellular, environmental and lifestyle related-signalling trigger the generation of mitochondrial ROS. As a consequence of this process, electrophilic aldehyde levels increase and bind to mitochondrial ETC elements, contributing to the persistence of the phenomenon. It is proposed that the self-perpetuating nature of this process reaches a threshold, leading to the loss of mitochondrial membrane potential, oxidative DNA damage, caspase activation, motility loss, phosphatidylserine externalisation, vitality loss and DNA fragmentation (see Figure 1) [75]. This perspective is consistent with the critical role of mitochondria as main ROS generator in male germ cells, which has been discussed in a previous section of this work.

### 5.1. Mitochondrial Dysfunction

Excessive mitochondrial ROS production is a milestone in the creation of the OS state that underlies male infertility. A crucial trait of this phenomenon is that it establishes a self-perpetuating, vicious cycle in which OS further promotes the generation of excessive ROS generation at a mitochondrial level. This self-perpetuating feature is enabled by the production of aldehydes such as acrolein, malondialdehyde and 4-hydroxynonenal generated by lipid peroxidation, which, in the first instance, carry out an oxidative attack on histidine, lysine and valine residues in mitochondrial proteins, particularly succinate dehydrogenase, an enzyme located in the inner mitochondrial membrane that participates in the ETC [76]. This leads to dysregulation of electron flow along the mitochondrial ETC and further production of ROS, perpetuating the cycle via a positive feedback circuit [54]. In this setting, any factor that breaks the redox balance of the male germ cell inducing OS is prone to trigger mitochondrial ROS generation and begin the previously mentioned cycle. These inducing factors include both exogenous ROS sources (smoking, obesity, radiation, pollution, etc.) and dietary patterns leading to a deficit in exogenous antioxidants (non-enzymatic antioxidants and antioxidant enzyme cofactors).

### 5.2. Lipid Peroxidation

Lipid peroxidation is a result of the oxidative attack of ROS over PUFAs, which are abundant molecules in spermatozoa membranes, particularly docosahexaenoic and arachidonic acids [77], since they are a factor that confers high fluidity to sperm plasma membranes, a crucial trait for fertilisation. Unfortunately, the chemical characteristics responsible for this trait, also make them a target for free radical attack and the induction of lipid peroxidation [39]. Once lipid peroxidation is initiated, lipid radicals react with adjacent carbons, leading to a peroxidation chain reaction. Quantification of lipid peroxidation is usually performed by measuring its end products. Levels of aldehydic products (malondialdehyde [MDA] and 4-hydroxyalkenals) are inversely correlated with sperm motility, viability, capacity for prolonged survival in vitro and competence for sperm-oocyte fusion [75]. This is supported by a study by Hosseinzadeh Colagar and colleagues [78], in which malondialdehyde levels (an end product of lipid peroxidation) were significantly lower in normospermic men than in men with asthenoteratozoospermia and oligoasthenoteratozoospermia and had negative correlation with sperm count, motility and morphology.

### 5.3. Apoptosis

As previously discussed, the self-perpetuating cycle of ROS generation at a mitochondrial level results in high levels of peroxidation, loss of mitochondrial membrane potential, membrane damage in the midpiece of the spermatozoa and decreased sperm motility [76,79]. Subsequently, caspase activation and phosphatidylserine exteriorization ensue, as shown by the activation of caspase 3 and annexin V binding following exposure to H_2_O_2_ [80]. This is consistent with a proposed ROS-mediated activation of MAPK pathways resulting in apoptosis in mice [81]. Further down this pathway, apoptosis in sperm starts to exhibit features different from those seen in somatic cell apoptosis. Although there is evidence of endonuclease activity (e.g., mitochondrial endonuclease G or cytosolic caspase-activated DNAse), these are unable to translocate to the nucleus due to the physical architecture of spermatozoa [39], since mitochondria and most of cytoplasm are concentrated in the midpiece, while nuclear DNA is located in the spermatozoon’s head.

Moreover, nuclear DNA sperm is found in a quasicrystalline state, making penetration by endonucleases highly unlikely [75], possibly taking part in perimortem DNA fragmentation after ROS-dependent membrane breakdown [82]. Furthermore, an endogenous nuclease has been identified in human spermatozoa, which shares with somatic cell nucleases a dependence on calcium and magnesium, thus making likely its involvement in the final stages of apoptosis [83].

The apoptotic process of sperm is critical in male infertility because of its close association to DNA damage, under the premise that apoptosis is initiated during spermatogenesis but the process is arrested due to the profound conformational changes of male germ cells following the meiotic process, which removes the apoptotic intracellular machinery. Consequently, DNA damage could also be attributed to an abortive apoptotic process [39,84].

### 5.4. DNA Damage and Sperm DNA Fragmentation

Spermatozoa are proposed to be particularly susceptible to DNA damage, given both a lesser degree of condensation for mitochondrial DNA and the absence of DNA repair systems for nuclear DNA [85]. Although most of the available evidence focuses on nuclear DNA damage in spermatozoa, it is worth noting that mitochondrial DNA damage has been associated with poor motility and reduced fertility [86].

Two main mechanisms for DNA damage in the context of male infertility have been described: endonuclease-mediated cleavage and ROS [75]. Interestingly, 8-hydroxy-2′-deoxyguanosine levels (8OHdG), a biomarker of oxidative DNA damage, has been found to be consistently elevated in the spermatozoa of infertile men. Simultaneously, DNA damage measured by terminal deoxynucleotidyl transferase dUTP nick end labelling (TUNEL) assay is strongly correlated to 8OHdG levels [87]. This correlation is highly indicative that DNA damage related to male infertility is primarily oxidative in origin [87,88].

Consistent with Aitkens’ hypothesis, an early stage in this process and probable triggering event seems to be the induction of O_2_^−^ generation in sperm mitochondria [79,89].

Types of DNA damage identified on spermatozoa include base mismatch, abasic sites, base modifications, DNA adducts and crosslink, pyrimidine dimers, single strand breaks and double strand breaks. All of the above can lead to SDF, and except for base mismatch and pyrimidine dimers, all are induced by ROS overproduction [90].

Indeed, ROS overproduction induces SDF among other deleterious effects [39]. Several mechanisms explain the induction of SDF by ROS: direct oxidative damage, lipid peroxidation by-products, and induction of abortive apoptosis. Direct oxidative damage to DNA bases is thought to be inflicted mainly by mitochondria-originated H_2_O_2_, which is able to penetrate the nucleus due to its low molecular weight and lack of electric charge [75,91]. Furthermore, by-products of lipid peroxidation such as MDA and 4-hydroxynonenal (4HNE) have been shown cause DNA damage through the introduction of DNA adducts such as 8OHdG, 1,N6-ethenoadenosine, and 1,N6-ethenoguanosine [92,93,94]. The relationship between ROS and apoptosis as a source of DNA damage has been described in the previous section.

Single strand breaks (SSB) and double strand breaks (DSB) are both main elements of SDF [90]. SSB are mainly a consequence of ROS-induced 8OHdG production, secondary to lipid peroxidation, mitochondrial and nuclear DNA base modifications [95,96]. 8OHdG destabilises DNA structure and produces DNA breaks [97]. SSB can present extensively at multiple points/regions of the genome and have been mainly associated with lower rates of clinical pregnancy or an increase in conception time [98]. Conversely, DSB can be a consequence of SSB during the replication process, ROS, and exogenous elements such as ionising radiation [99,100]. DSB have been associated with an increased risk of miscarriage, decreased embryo quality and lower successful implantation rates following the use of assisted reproduction techniques (ART) such as intracytoplasmic sperm injection (ICSI) [98].

There are several methods available for the evaluation of sperm DNA fragmentation/integrity including: TUNEL assay [101], sperm chromatin structure assay (SCSA) [102], and sperm chromatin dispersion (SCD) [103]. Nevertheless, the above assays are unable to discriminate SSB from DSB [104], unlike the Comet assay (particularly the alkaline and two-tailed methodologies), which is capable of discriminating between SSB and DSB [105,106].

Regarding the effects of DNA damage and sperm DNA fragmentation on human reproduction, SDF has been inversely associated with probability of natural pregnancy in a Danish cohort [107]. Furthermore, SDF becomes particularly relevant in the clinical setting of ART. Despite contradictory evidence [108], possibly owing to differences between SDF detection assays and other confounding factors, a high SDF value is associated with decreased pregnancy rate with IUI and IVF and with increased miscarriage rate following both IVF and ICSI [90]. All of the above is consistent with the correlation between high SDF levels and overall worse reproductive outcomes [24,25,26].

The current management of SDF and OS in unexplained infertility focuses on the use of several interventions, with a variable degree of supporting evidence: antioxidant supplementation [109,110], lifestyle modifications (such as weight loss and smoking cessation) [111,112,113], recurrent ejaculation [114], and ultimately, the use of sperm processing and preparation preceding ICSI [90].

## 6. Proposal for a Novel Antioxidant Therapeutic Strategy

Taking into account the specific intracellular phenomena and sensitive sperm-development timeframes, we hypothesise that positive achievements will result from interventions based on the relationship between the time course of stages of spermatogenesis and OS. Thus, these processes could be favoured throughout the spermatogenesis cycle by the pertinent effect of appropriate time-related pharmacological agents in order to improve the clinical outcomes of male infertility.

Aiming to properly contextualise the reasoning underlying our proposal, key aspects of sperm development are described below:

Spermatogenesis, the process by which spermatogonial cells become spermatozoa, is a complex process occurring within seminiferous tubules of the testis whose exhaustive description is beyond the aim of this work but can be broadly divided into four phases: (1) mitosis; (2) meiosis; (3) spermiogenesis and (4) spermiation [115]. In humans, a full spermatogenesis cycle lasts approximately 74 days [116], varying from 42 to 76 days [117], with a new cycle initiating every 16 days. At any given moment, 6 spermatogenesis cycles are taking place in each seminiferous tubule, each one in a particular phase. These phases are described below:

(1) Mitosis: Progenitor A_dark_- and A_pale_-spermatogonia undergo continuous mitosis for renewal of the germ cell line, while Apale-spermatogonia commit to differentiation and divide to form B-spermatogonia, subsequently dividing to form preleptotene spermatocytes [118].
(2) Meiosis: Diploid spermatocytes (2n2c) undergo 2 meiotic divisions to form haploid spermatids (nc). Primary spermatocytes are the first cells to undergo meiosis [119]. During meiotic division, chromosomal recombination and DNA exchange through crossing-over ensure genetic diversity of these cells from their adult precursors.
(3) Spermiogenesis: Consists of the transformation of round spermatids into elongated, mature spermatozoa. It includes the loss of cytoplasm, migration of cytoplasmic organelles, formation of the acrosome from the Golgi apparatus, formation of the flagellum from the centriole, nuclear compaction to about 10% of former size, and reorganisation of mitochondria around the sperm midpiece [119]. Moreover, the spermatid nucleus undergoes remodelling and condensation, associated with the displacement of histones by transition proteins and then by protamines [120]. Consequently, the vast majority of sperm DNA is coiled into toroids by protamines, with a significantly lower fraction remaining bound to histones, and the DNA is attached to the sperm nuclear matrix at MARs (matrix attachment regions) at medium intervals of roughly 50 kb throughout the genome [121]. It is during this process that germ cells are most sensitive to OS [39]. This could be explained by a number of factors, such as the limited glutathione replenishment and DNA repairing capacities of spermatids [122]. This inherent susceptibility is compensated by the protective antioxidant role of Sertoli cells, mainly exerted through the activity of SOD, as well as reductase, transferase and peroxidase [67].
(4) Spermiation: The mature spermatozoon is freed of its anchorage to the Sertoli cell, being released into the tubule lumen.

**Figure 2:**
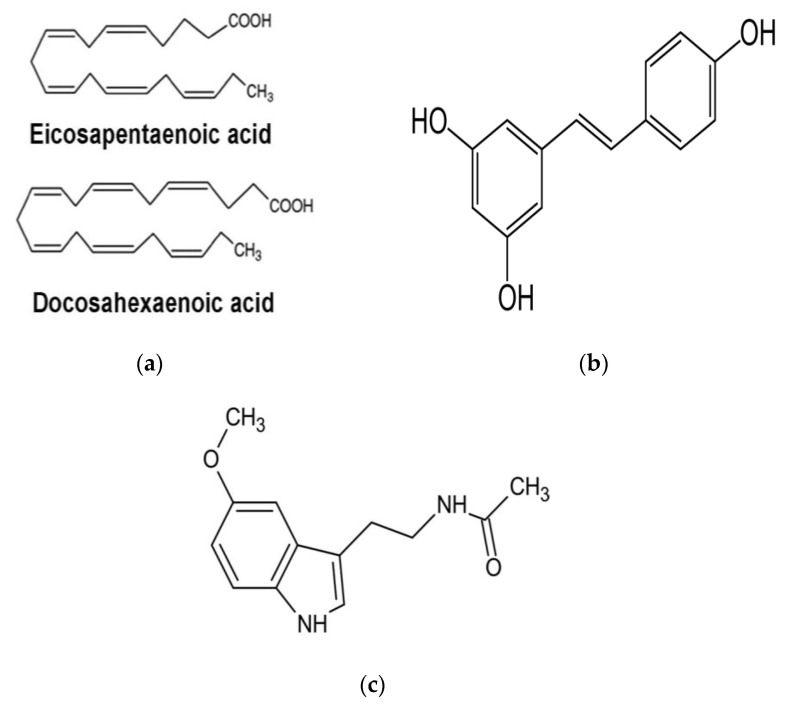
Proposed antioxidant regimen: (**a**) Long chain ω-3 polyunsaturated fatty acids; (**b**) Resveratrol; (**c**) Melatonin.

Our proposal includes 3 antioxidants (see Figure 2), whose respective properties are described below:

Omega-3 (ω-3) polyunsaturated fatty acids. PUFAs comprise five substances: eicosapentaenoic acid (EPA), alpha-linolenic-acid, stearidonic acid, docosahexaenoic acid (DHA), and docosapentaenoic acid. Of particular relevance are long-chain ω-3 PUFAs such as EPA and DHA, which are abundant in the body lipids of fatty fish, the liver of white lean fish, and the blubber of marine mammals [123].

EPA and DHA are highly susceptible to free-radical oxidation [124], yet they have been shown to reduce urinary F2-isoprostane levels (a lipid peroxidation biomarker) and enhance the antioxidant systems of the cell [125]. Gao and colleagues published an in vitro study which showed that oxidised EPA and DHA react directly with Keap1, a negative regulator of Nrf2, inducing Nrf2-directed gene expression [125], thus acting as indirect antioxidants.

The consumption of long chain ω-3 PUFAs has shown improvements in sperm SOD and CAT activities [126], sperm cell concentration [126], sperm motility [127] and DNA fragmentation [128]. These compounds have been safely used in doses of up to 1.8 g/day in a RCT [126].

Resveratrol. Resveratrol is a phenolic compound derived from stilbene naturally found in foods such as grapes, nuts, cranberries and red wine [129]. It possesses several beneficial effects on human health that are derived from its antioxidant and anti-inflammatory properties [130].

The antioxidant properties that resveratrol exhibits are partly due to being an excellent scavenger of ·OH, O_2_^−^ and metal-induced ROS, thereby protecting cell membranes from lipid peroxidation and DNA from ROS-mediated damage [131].

Moreover, this compound has been shown to act through the transcription factor named nuclear factor erythroid 2–related factor 2 (Nrf2) pathway, as it downregulates Keap1 [132], a protein involved in the ubiquitination and proteasomal degradation of Nrf2 through its binding and secondary retention in the cytoplasm [133]. Consequently, it increases the expression and translocation to the nucleus of Nrf2, which binds to antioxidant response elements (AREs) in the promoter regions of genes encoding cytoprotective proteins and antioxidant enzymes such as catalase, superoxide dismutase, glutathione peroxidase, NADPH quinone oxidoreductase and glutathione-S-transferase [134]. This mechanism has been identified in hepatocytes [135], lung epithelial cells [136] and endothelial cells [137].

Furthermore, the upregulation of the Nrf2 pathway by resveratrol could be important in the maintenance of mitochondrial homeostasis and structural integrity [138]. The above becomes particularly relevant given the key role that mitochondrial ROS production appears to have in idiopathic male infertility.

Interestingly, resveratrol was also found to have a significant inhibitory effect on the NF-κB signalling pathway after cellular exposure to metal-induced radicals [131].

In 2020, Illiano et al. treated 20 patients with idiopathic infertility with a resveratrol-based multivitamin supplement containing a dose of 150 mg of resveratrol daily, for 6 months, after which they showed improved sperm motility and concentration compared to baseline measurements [139]. Limitations to the study include small sample size, lack of control group and the absence of measurements related to OS or antioxidant activity. Nevertheless, it sets a precedent regarding a beneficial effect of the compound in this particular clinical setting.

Melatonin. Melatonin is an ancient molecule with presence throughout Eukarya and Bacteria domains, and first appeared as a way to mitigate ROS production secondary to aerobic metabolism [140]. Since its discovery in plants in 1995, it has been identified in a variety of foods and medicinal plants [140,141].

It exhibits antioxidant activity through various direct and indirect mechanisms: ROS scavenging [142,143,144], transition metal binding [145,146], stimulating the activity of antioxidant enzymes such as SOD (particularly isoforms SOD1 and SOD2), catalase and glutathione peroxidase [147], which could be explained by its role in promoting the expression of Nrf2 [148]. Interestingly, its metabolites can also exert similar functions [149,150], suggesting that the antioxidant properties of melatonin continue beyond its metabolism [151].

Melatonin is an amphiphilic molecule, with the capacity to cross biological membranes and reach any cellular and subcellular compartment, owing to this property its high rate of distribution and the ability to exert its antioxidant activity [142,152]. The above translates into a protective effect against the attack of ROS over lipids [153,154], proteins [155,156] and DNA [157,158]. Furthermore, its transport could involve more than passive diffusion, as transport systems located in mitochondria, such as facilitative glucose transporters GLUT/SLC2A and proton-driven oligopeptide transporter PEPT1/2, have been shown to have an active role in facilitating melatonin transport across membranes [159].

Moreover, studies in mice showed melatonin’s ability to reverse the effects of cyanide, an ETC Complex IV inhibitor [160]. Furthermore, it showed a protective role against the effect of several neurotoxins over ETC Complex I. These actions could not be replicated through the use of vitamin C or E [161].

Furthermore, in recent work Malmir and colleagues studied the in vitro effect of melatonin over spermatozoa collected from men with idiopathic asthenoteratozoospermia. DNA fragmentation measured by TUNEL, sperm chromatin dispersion and malondialdehyde levels were significantly reduced while mitochondrial membrane potential and viability were significantly increased in the melatonin group compared to control [162].

Given its stability during digestion and fast absorption, melatonin possesses a high bioavailability [151]. Oral administration has been reported to be safe in humans at doses up to 1000 mg daily for one month [163,164,165].

**Figure 3:**
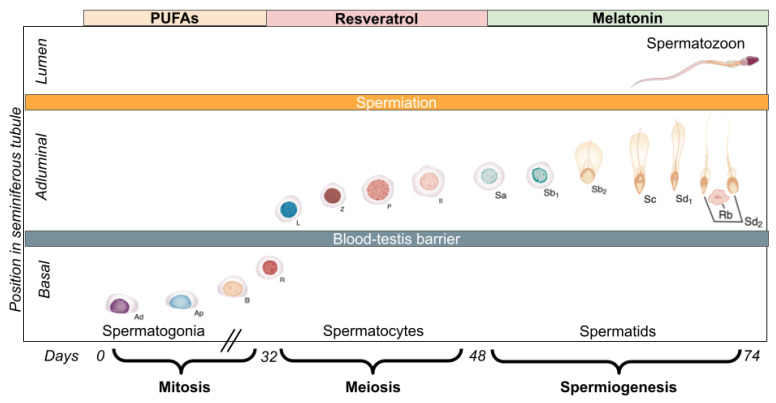
Timeframes in human spermatogenesis and proposed antioxidant intervention. Ad, Dark type A spermatogonium; Ap, pale type A spermatogonium; B, type B spermatogonium; II, secondary spermatocyte; L, leptotene spermatocyte; P, pachytene spermatocyte; R, resting or preleptotene primary spermatocyte; Rb, residual body; Sa (a), Sb1 (b1), Sb2 (b2), Sc (c), Sd1 (d1), Sd2 (d2), spermatids; Z, zygotene spermatocyte. (Adapted from Servier Medical Art; Campbell-Walsh-Wein Urology, Part VI: Reproductive and Sexual Function, page 1398; and Henry TD, Porucznik CA, Honda TJ et al. Differential impacts of particulate air pollution exposure on early and late stages of spermatogenesis. Ecotoxicol Environ Saf. 2021; 220:112419).

These compounds share a few characteristics that might be relevant to the effectiveness of the regimen: they exert their antioxidant capacities through various mechanisms, many of which overlap. In addition, the three of them have been shown to promote the activity of intrinsic, enzymatic antioxidant systems through the Nrf2 pathway. In the case of PUFAs, the aim of their use during the mitotic phase of spermatogenesis is to generate non-hypoxic preconditioning of male germ cells in order to strengthen their enzymatic antioxidant defences. Moreover, we propose the use of resveratrol during meiosis because of its simultaneous role as direct and indirect antioxidant, aiming to neutralise the possible exogenous ROS before the most vulnerable moment of spermatogenesis: spermiogenesis. Furthermore, melatonin is the antioxidant proposed for the spermiogenesis phase due to the following features: (i) multiple antioxidant capabilities and mitochondrial stabilising properties; (ii) ample distribution throughout the cell given its amphiphilic nature and the proposed active transport at a mitochondrial level; and (iii) prolonged effect due to the analogous actions of its active metabolites. This proposal (graphically presented in Figure 3) intends to take into account the temporality of the spermatogenesis process, albeit in vivo several cycles of spermatogenesis are taking place simultaneously, as previously described.

## 7. Concluding Remarks and Future Perspectives on Combined Antioxidant Therapy against Male Infertility

As we have established, several reports support the association of high seminal ROS levels both with impaired sperm parameters [166,167] and seminal DNA fragmentation [168,169,170].

Therefore, the use of antioxidants as a treatment for male infertility presents a reasonable and biologically plausible intervention. Despite how promising it seems, few studies have been able to prove a consistent benefit of antioxidant-based therapy by themselves and evidence is largely controversial (see Appendix A, Table A1). Furthermore, available evidence is often low-quality: a 2019 Cochrane review of 61 studies showed that antioxidants may improve live birth and clinical pregnancy rates, although with concerns regarding quality evidence and high risk of bias. Moreover, antioxidant versus antioxidant comparisons were not performed, as insufficient studies compared the same interventions [171]. Indeed, there is no clear consensus regarding the optimal components and structure of an antioxidant treatment regimen [1].

It is worth taking into account that for most RCT and other trials using antioxidant treatment for male infertility, there is considerable variability regarding sample selection and general inclusion criteria. Despite the amount of evidence supporting OS as a core element in male infertility pathophysiology, idiopathic male infertility cohorts are highly heterogeneous in terms of the specific abnormalities in spermiogram and other clinical evidence that leads to their inclusion in this group. In other words, the concept of idiopathic male infertility becomes an umbrella term for a myriad of clinical conditions that lead to dysfunction of male reproductive function.

A necessary approach to improve the outcomes in future therapeutic interventions could be to effectively establish OS is in fact a main factor in the pathophysiology of their particular reproductive dysfunction by adding high OS biomarker levels as an inclusion criteria for the use of an antioxidant regimen on idiopathic male infertility. Regarding measurements and outcomes, the determination of said biomarkers during and after the intervention becomes mandatory along with sperm quality and in vivo fertility follow-ups in order to establish consistent correlations.

Concerning the components and structure of the antioxidant regimen itself, there is still need for further evidence on the role that commonly cited antioxidants have under in vivo conditions in human sperm. Nevertheless, given the fact that male fertility is a continuous, highly complex process with an equally nuanced pathophysiology, future antioxidant regimens should entail a dynamic, longitudinal approach to antioxidant compound selection and timing of interventions.

## Data Availability

Not applicable.

## Appendix A

**Table A1:** Studies regarding the use of antioxidants for male infertility. RCT: randomized controlled trial; TAC: total antioxidant capacity.

Authors	Journal	Population	*N*	Antioxidant	Follow-Up (Months)	Outcomes	Study Design
Steiner et al., 2020 [172]	*Fertility and Sterility*	Oligozoospermia and/or asthenozoospermia and/or teratozoospermia or DNA fragmentation >25%	174	500 mg vitamin C 400 mg vitamin E 0.20 mg selenium 1000 mg l-carnitine 20 mg zinc 1000 μg folic acid 10 mg of lycopene, daily for 3–6 months	6	Improvement in sperm concentration No improvement in sperm morphology, motility or DNA fragmentation. No improvement over in vivo pregnancy or live-birth rates	RCT
Jannatifar et al., 2019 [173]	*Reproductive Biology and Endocrinology*	Oligoasthenoteratozoospermia	50	600 mg NAC daily for 3 months	3	Improvement in sperm count, morphology, motility, DNA fragmentation and protamine deficiency	Clinical trial (no control group)
Alahmar & Sengupta, 2020 [174]	*Biological Trace Element Research*	Oligoasthenoteratospermia	70	200 mg CoQ10 or 200 μg selenium daily for 3 months	3	Improvement in sperm count, progressive sperm motility, total sperm motility, TAC and SOD in both groups	RCT
Stenqvist et al., 2018 [175]	*Andrology*	Men from infertile couples, with normal testosterone, LH and FSH levels and DNA fragmentation ≥25%	77	30 mg vitamin C 5 mg vitamin E 0.5 μg vitamin B12 750 mg L-carnitine 10 mg CoQ10 100 μg folic acid 5 mg zinc 25 μg selenium	6	No improvement on DNA fragmentation or standard semen parameters	RCT
Kessoupoulou et al., 1995 [176]	*Fertility and Sterility*	Men with increased ROS levels measured through chemiluminescent method	30	600 mg vitamin E daily for 6 months	3	No improvement in semen parameters, improvement in in vitro zona pellucida binding	RCT
Hawkes et al., 2009 [177]	*Journal of Andrology*	Normozoospermia	42	300 μg selenium daily for 12 months	24	No improvement in spermiogram parameters. Se status of testes appears unresponsive to dietary Se intake	RCT
Moilanen et al., 1993 [178]	*International Journal of Andrology*	Asthenozoospermia or oligoasthenozoospermia	15	300 mg vitamin E daily for 3 months	3	No improvement in sperm parameters or pregnancy rates	RCT
Greco et al., 2005 [110]	*Journal of Andrology*	Unexplained infertility and ≥15% DNA fragmentation	64	1 g vitamin C 1 g vitamin E daily for 2 months	2	No improvement in spermiogram parameters	RCT
Rolf et al., 1999 [179]	*Human Reproduction*	Asthenozoospermia with/without oligozoospermia	31	1 g vitamin C 800 mg vitamin E daily for 2 months	2	No improvement in spermiogram parameters, no pregnancy	RCT
Sigman et al., 2006 [180]	*Fertility and Sterility*	Asthenozoospermia	21	2 g L-carnitine 1 g L-acetyl-carnitine daily for 4 months	6	No improvement in spermiogram parameters	RCT
